# Automated detection of colorectal tumors based on artificial intelligence

**DOI:** 10.1186/s12911-020-01314-8

**Published:** 2021-02-01

**Authors:** Kwang-Sig Lee, Sang-Hyuk Son, Sang-Hyun Park, Eun Sun Kim

**Affiliations:** 1 AI Center, Korea University College of Medicine, Seoul, Korea; 2 Storage Solution for Doctors Co., Ltd., Seoul, Korea; 3 Biomedical Research Institute, Korea University College of Medicine, Seoul, Korea; 4 Department of Gastroenterology, Korea University College of Medicine, University Anam Hospital, 73 Goryeodae-ro, Seongbuk-gu, Seoul, 02841 Korea

**Keywords:** Colon, Neoplasm, Artificial intelligence

## Abstract

**Background:**

This study developed a diagnostic tool to automatically detect normal, unclear and tumor images from colonoscopy videos using artificial intelligence.

**Methods:**

For the creation of training and validation sets, 47,555 images in the jpg format were extracted from colonoscopy videos for 24 patients in Korea University Anam Hospital. A gastroenterologist with the clinical experience of 15 years divided the 47,555 images into three classes of Normal (25,895), Unclear (2038) and Tumor (19,622). A single shot detector, a deep learning framework designed for object detection, was trained using the 47,255 images and validated with two sets of 300 images—each validation set included 150 images (50 normal, 50 unclear and 50 tumor cases). Half of the 47,255 images were used for building the model and the other half were used for testing the model. The learning rate of the model was 0.0001 during 250 epochs (training cycles).

**Results:**

The average accuracy, precision, recall, and F1 score over the category were 0.9067, 0.9744, 0.9067 and 0.9393, respectively. These performance measures had no change with respect to the intersection-over-union threshold (0.45, 0.50, and 0.55). This finding suggests the stability of the model.

**Conclusion:**

Automated detection of normal, unclear and tumor images from colonoscopy videos is possible by using a deep learning framework. This is expected to provide an invaluable decision supporting system for clinical experts.

## Background

Colorectal cancer is a leading cause of disease burden in the world. It was the third and second greatest sources of cancer incidence and mortality in the world for year 2018, respectively—it accounted for 10.2% (1,849,518) of new cancer cases (18,078,957) and 9.2% (880,792) of total cancer deaths (9,555,027) [1, 2]. This global pattern is consistent with its local counterpart in Korea. Colorectal cancer ranked second and third in terms of cancer incidence and mortality in the country for year 2016, respectively—it was responsible for 12.3% (28,127) of new cancer cases (229,180) and 10.7% (8,358) of total cancer deaths (78,194) [3]. Indeed, its economic burden became more significant in the country during 2000–2010. Its ranking and amount registered a rapid rise from the 5^th^/837 in 2000 to the 3^rd^/2,210 in 2010 (million US$ for the total population) [4]. Colonoscopy is an effective way to screen colorectal tumors and prevent colorectal cancer [5, 6]. However, its performance depends on various factors including tumor size and screening conditions. Its sensitivity can be as low as 0.75 depending on tumor size [5, 6]. This situation gets even worse with image blurring from screen shaking or fluid injection. However, the recent development of artificial intelligence (AI) is expected to provide an invaluable decision supporting system for endoscopists to overcome this challenge.

The artificial neural network is a popular AI model including one input layer, one, two or more hidden layers and one output layer. Neurons in a previous layer unite with the weights in the next layer. This process can be denoted as the feedforward algorithm. Then, these weights are refined by the amounts of their contributions for a difference between the actual and predicted final outputs. This process can be denoted as the backpropagation algorithm. These processes are iterated until a certain criterion is met for the accurate prediction of the dependent variable [7, 8]. The convolutional neural network (CNN) is an artificial neural network including convolutional layers. In the convolutional layer, a feature detector slides across input data and the dot product of its elements and their input data counterparts is computed. This process leads to effective identification of the CNN for specific features of the input data [8, 9]. Based on a recent review, the CNN is expected to aid in endoscopists’ accurate diagnosis of gastrointestinal regions [10]. Especially, some studies report that the CNN outperformed endoscopists for the classification of colorectal tumors (86% vs. 74%) [11, 12]. However, little study has been done and more effort is needed on this topic. In this context, this study developed a diagnostic tool to automatically detect normal, unclear and tumor images from colonoscopy videos using the CNN.

## Methods

### Study participants

This study was approved by the Institutional Review Board (IRB) of Korea University Anam Hospital on October, 17, 2019 (IRB No. 2019AN0424). Informed consent was waived by the IRB. For the creation of training and validation sets, 47,555 images in the jpg format were extracted from colonoscopy videos for 24 patients in Korea University Anam Hospital (Additional file 1: Table S1). The resolution of a video was 720 × 480 i60. A gastroenterologist with the clinical experience of 15 years divided the 47,555 images into three classes of Normal (25,895), Unclear (2038) and Tumor (19,622). The class of Unclear included blurred images with screen shaking, fluid injection and other causes.

### Model development

A single shot detector (SSD) [9, 13], a deep learning framework designed for object detection, was trained using the 42,555 images and validated with two sets of 300 images—each validation set included 150 images (50 normal, 50 unclear and 50 tumor cases). Half of the 47,255 images were used for building the model and the other half were used for testing the model. This process of model building and testing (“training process”) was repeated 250 times (250 epochs) to improve the model. The two validation sets with 300 images total, were completely separate from the training process and were used for validating the model. Here, the training set of 47,255 images and one validation set of 150 images (validation set 1) came from 5 patients while the other validation set of 150 images (validation set 2) came from other 19 patients (Table S1, supplementary information). The learning rate of the model was 0.0001 during the 250 epochs. SSD, which does not require the stages of proposal generation and feature resampling, is faster than another detection model, Faster R-CNN [14]. Indeed, SSD has an important advantage as compared to CNNs for disease classification. These models only classify a single disease, i.e., testing whether it belongs to a certain category (e.g., normal vs. tumor). They do not provide additional information on the regions of interest (i.e., the locations of the lesions). On the contrary, SSD output covers not only the types of various diseases but also the locations of their lesions, which helps clinicians improve their diagnostic criteria.

### Performance measures

Accuracy, precision, recall, and F1 score are the performance measures of the model [9]. These measures were calculated for three thresholds of the intersection over union (IOU), i.e., 0.45, 0.50, and 0.55 [9, 15]. The model can be considered stable when its performance measures show no or little changes with respect to the three IOU thresholds. The Python programming language (v.3.52) and a graphics card (GeForce GTX 1080 Ti D5X 11 GB) were used for the analysis.

## Results

**Fig. 1:**
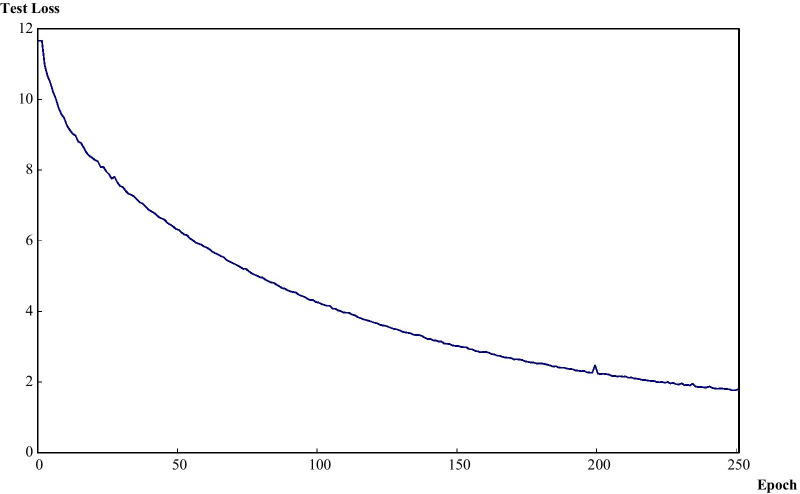
Test loss during training epochs

**Fig. 2:**
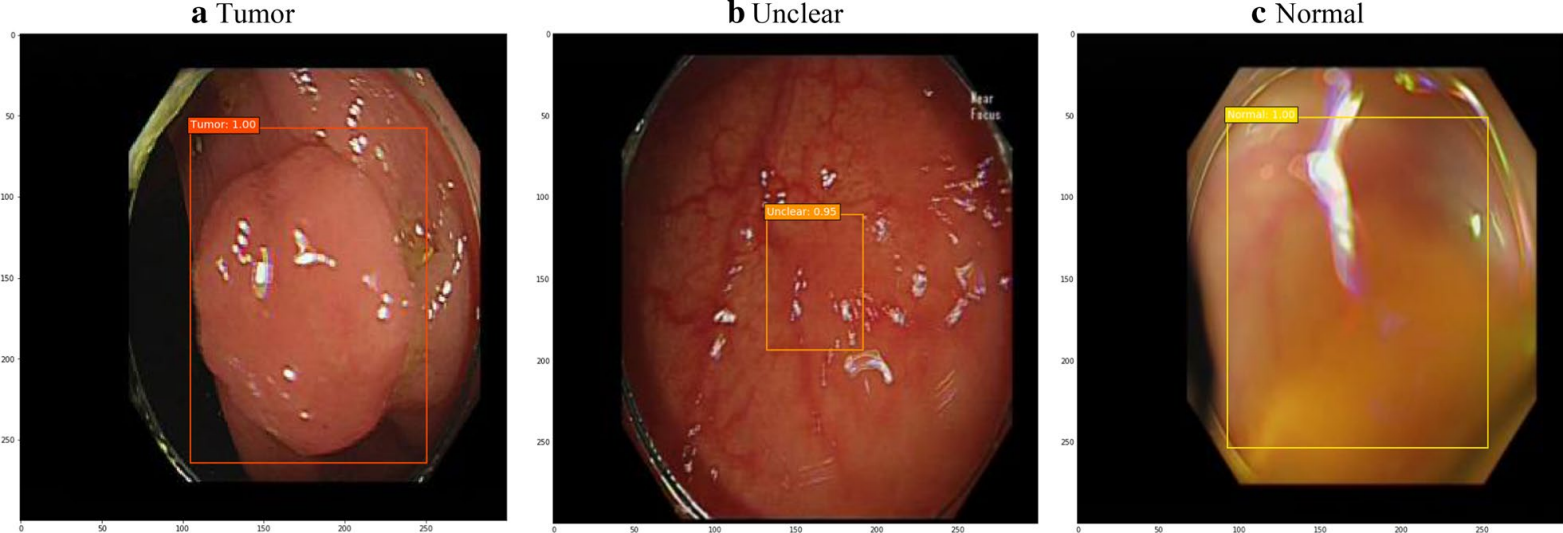
Examples of predicted classes and boxes. **a** Tumor, **b** unclear and **c** normal

After 250 training epochs, the test loss of the model decreased from 11.66 to 1.79 (Fig. 1). Table 1 shows the confusion matrix of the model, which compares the predicted classes against the true classes for 150 images in each of the validation sets 1 and 2. The prediction was repeated 10 times, and the average over 10 runs is presented in Table 1. The confusion matrix had no change with respect to the IOU threshold (0.45, 0.50, and 0.55). This finding suggests the stability of the model. The accuracy, precision, recall, and F1 score of the model are shown in Table 2. These values were derived from Table 1, which represents the confusion matrix of the model with the average over 10 runs of the prediction. These performance measures also had no change with respect to the IOU threshold (0.45, 0.50, and 0.55). The respective accuracy measures of the validation sets 1 and 2 were 0.9733 and 9067. The respective precision results of the validation sets 1 and 2 were: (1) 1.0000 and 1.0000 for Normal, (2) 1.0000 and 1.0000 for Unclear, (3) 1.0000 and 0.9231 for Tumor and (4) 1.0000 and 0.9744 for the average of the three groups. The respective recall measures of the validation sets 1 and 2 were: (1) 1.0000 and 0.9800 for Normal, (2) 1.0000 and 0.7800 for Unclear, (3) 0.9200 and 0.9600 for Tumor and (4) 0.9733 and 0.9067 for the average of the three groups. Similarly, the respective F1 scores of the validation sets 1 and 2 were: (1) 1.0000 and 0.9899 for Normal, (2) 1.0000 and 0.8764 for Unclear, (3) 0.9583 and 0.9412 for Tumor and (4) 0.9865 and 0.9393 for the average of the three groups. Both the validation sets 1 and 2 were completely separate from the training process and the unit of analysis is a normal, unclear or tumor image (not a patient). For this reason, both the validation sets 1 and 2 were used for validating the model in this study. However, the validation set 1 came from 5 patients (the sources of the training set of 47,255 images as well), while the validation set 2 came from other 19 patients (Table S1, supplementary information). The validation set 2 is expected to be more reliable than the validation set 1. Examples of correctly classified cases are presented in Fig. 2.

**Table 1:** Confusion matrix

*Predicted*	*True*			
	Background	Normal	Unclear	Tumor
Validation set 1 (5 patients)				
Background^a^	0	0	0	4
Normal	0	50	0	0
Unclear	0	0	50	0
Tumor	0	0	0	46

*Predicted*	*True*			
	Background	Normal	Unclear	Tumor
Validation set 2 (19 patients)				
Background	0	1	7	2
Normal	0	49	0	0
Unclear	0	0	39	0
Tumor	0	0	4	48

^a^Background indicates that the model does not bring any detection result

**Table 2:** Model performance

	Validation set 1	Validation set 2
Accuracy	0.9733	0.9067
Normal		
Precision	1.0000	1.0000
Recall	1.0000	0.9800
F1 score	1.0000	0.9899
Unclear		
Precision	1.0000	1.0000
Recall	1.0000	0.7800
F1 score	1.0000	0.8764
Tumor		
Precision	1.0000	0.9231
Recall	0.9200	0.9600
F1 score	0.9583	0.9412
Class average		
Precision	1.0000	0.9744
Recall	0.9733	0.9067
F1 score	0.9865	0.9393

## Discussion

In this study, 47,555 images were extracted from colonoscopy videos for 24 patients in a general hospital, and a deep learning framework (SSD) was developed to automatically detect normal, unclear and tumor images. The performance of the model was excellent in standard measures. The average accuracy, precision, recall, and F1 score over the category were 0.9067, 0.9744, 0.9067 and 0.9393, respectively. These performance measures had no change with respect to the IOU threshold (0.45, 0.50, and 0.55). This finding suggests the stability of the model. A recent review shows that the development and application of the CNN has been popular and successful in gastrointestinal endoscopy with the range of its accuracy from 75.1 to 94.0% [10]. Specifically, the CNN was reported to be better than endoscopists for the classification of colorectal tumors, that is, 86% versus 74% in terms of accuracy [11, 12]. But it has been very rare in this area to develop and apply SSD with multiple classes such as normal, unclear and tumor images. In this vein, this study developed a diagnostic tool to automatically detect normal, unclear and tumor images from colonoscopy videos using SSD. The performance of SSD with three classes in this study was comparable to the very best of the existing literature with binary classes.

**Fig. 3:**
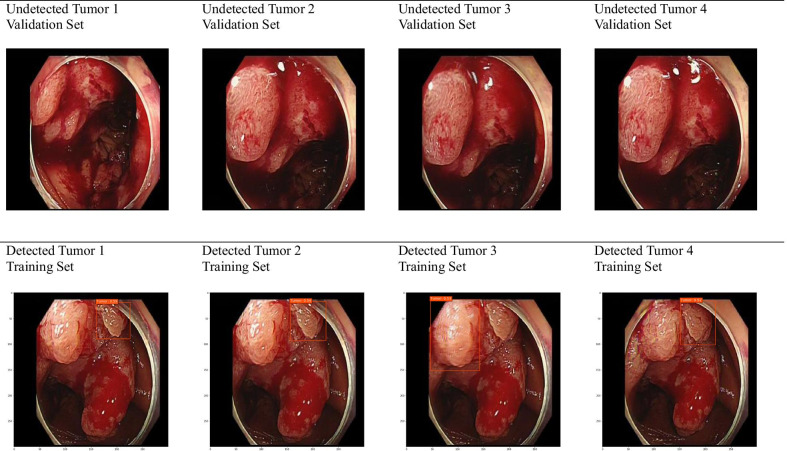
Undetected and detected tumors

However, this study had some limitations. Four undetected tumors in the validation set 1 are displayed in the first row of Fig. 3 (These tumors were classified as background with no detection results). For comparison, four detected tumors from the training set are presented in the second row. The undetected tumors in the validation set look bigger and more evenly spread than do their detected counterparts from the training set. The former look more homogenous than do the latter in terms of color as well. This would explain why the model predicted the undetected tumors to be background with no detection result. One effective solution would be to expand the training set with this type of tumors and to perform additional training of the model. Indeed, it would be a good topic for future research to diversify the classes of colonoscopy images in terms of tumor’s shape, color and severity.

## Conclusion

Automated detection of normal, unclear and tumor images from colonoscopy videos is possible by using a deep learning framework. This is expected to provide an invaluable decision supporting system for clinical experts.

## Supplementary information

**Additional file 1: Table S1.** Descriptive statistics of study participants.

## Data Availability

The datasets used and/or analysed during the current study are available from the corresponding author on reasonable request.

## Abbreviations

AI: Artificial Intelligence
CNN: Convolutional Neural Network
SSD: Single Shot Detector
